# Supplementary material for: End Sequence Analysis Toolkit (ESAT) expands the extractable information from single-cell RNA-seq data
Source: Genome Res. 2016 Oct;26(10):1397–410. doi: 10.1101/gr.207902.116 (PMC5052061; doi:10.1101/gr.207902.116)
Supplement: Supplemental Material [file supp_gr.207902.116_Supp_Materials.tar.gz › ESAT-esat_v0.1/doc/html/classbroad_1_1core_1_1sequence_1_1_fasta_quality_binary_i_o.html]

ESAT: broad.core.sequence.FastaQualityBinaryIO Class Reference


ESAT


- Main Page
- Related Pages
- Packages
- Classes
- Files

- Class List
- Class Index
- Class Hierarchy
- Class Members

All Classes Namespaces Files Functions Variables Enumerator Pages

- broad
- core
- sequence
- FastaQualityBinaryIO

Public Member Functions |
Static Public Attributes |
List of all members

broad.core.sequence.FastaQualityBinaryIO Class Reference

|  |  |
| --- | --- |
| Public Member Functions | |
| void | createBinaryFromAscii (InputStream in, String outFile) throws IOException |
|  | |
| void | toDBFromAscii (InputStream in, final Connection conn, String table) throws IOException, SQLException |
|  | |
| void | createAsciiFromBinary (String binaryQualityFile, int numQualBasesPerRow) throws IOException |
|  | |
| int[] | getQualityBases (Connection conn, String table, String seqId, int start, int numOfBases) throws SQLException |
|  | |
| int[] | getQualityBases (Connection conn, String table, String seqId) throws SQLException |
|  | |

|  |  |
| --- | --- |
| Static Public Attributes | |
| static final int | SEQ\_ID\_REC\_LENGTH = 300 |
|  | |
| static final int | BUFFER\_SIZE = 8192 |
|  | |

## Member Function Documentation

|  |  |  |  |
| --- | --- | --- | --- |
| void broad.core.sequence.FastaQualityBinaryIO.createAsciiFromBinary | ( | String | *binaryQualityFile*, |
|  |  | int | *numQualBasesPerRow* |
|  | ) |  | throws IOException |

|  |  |  |  |
| --- | --- | --- | --- |
| void broad.core.sequence.FastaQualityBinaryIO.createBinaryFromAscii | ( | InputStream | *in*, |
|  |  | String | *outFile* |
|  | ) |  | throws IOException |

Here is the call graph for this function:

|  |  |  |  |
| --- | --- | --- | --- |
| int [] broad.core.sequence.FastaQualityBinaryIO.getQualityBases | ( | Connection | *conn*, |
|  |  | String | *table*, |
|  |  | String | *seqId*, |
|  |  | int | *start*, |
|  |  | int | *numOfBases* |
|  | ) |  | throws SQLException |

Retrieves quality base calls for a given sequence from a binary quality assembly file.

Parameters
:   |  |  |
    | --- | --- |
    | Table | - Database table containing the assembly quality info. |
    | seqId | - Sequence id of interest. |
    | start | - Zero based start of base (included) |
    | numOfBases | - number of bases to retrieve starting from base start. |

Returns

Exceptions
:   |  |  |
    | --- | --- |
    | SQLException |  |

|  |  |  |  |
| --- | --- | --- | --- |
| int [] broad.core.sequence.FastaQualityBinaryIO.getQualityBases | ( | Connection | *conn*, |
|  |  | String | *table*, |
|  |  | String | *seqId* |
|  | ) |  | throws SQLException |

Retrieves quality base calls for a given sequence from a binary quality assembly file. Use wisely, it returns an integer array with ALL quality scores.

Parameters
:   |  |  |
    | --- | --- |
    | Table | - Database table containing the assembly quality info. |
    | seqId | - Sequence id of interest. |

Returns

Exceptions
:   |  |  |
    | --- | --- |
    | SQLException |  |

|  |  |  |  |
| --- | --- | --- | --- |
| void broad.core.sequence.FastaQualityBinaryIO.toDBFromAscii | ( | InputStream | *in*, |
|  |  | final Connection | *conn*, |
|  |  | String | *table* |
|  | ) |  | throws IOException, SQLException |

## Member Data Documentation

final int broad.core.sequence.FastaQualityBinaryIO.BUFFER\_SIZE = 8192

static

final int broad.core.sequence.FastaQualityBinaryIO.SEQ\_ID\_REC\_LENGTH = 300

static

---

The documentation for this class was generated from the following file:

- java/broad/core/sequence/FastaQualityBinaryIO.java


---

Generated on Fri Aug 15 2014 15:10:55 for ESAT by  

 1.8.7
